# Sequence variation in mature microRNA-608 and benefit from neo-adjuvant treatment in locally advanced rectal cancer patients

**DOI:** 10.1093/carcin/bgw073

**Published:** 2016-07-05

**Authors:** Francesco Sclafani, Ian Chau, David Cunningham, Andrea Lampis, Jens Claus Hahne, Michele Ghidini, Hazel Lote, Domenico Zito, Josep Tabernero, Bengt Glimelius, Andres Cervantes, Ruwaida Begum, David Gonzalez De Castro, Sanna Hulkki Wilson, Clare Peckitt, Zakaria Eltahir, Andrew Wotherspoon, Diana Tait, Gina Brown, Jacqueline Oates, Chiara Braconi, Nicola Valeri

**Affiliations:** ^1^Department of Medicine, The Royal Marsden NHS Foundation Trust, Surrey SM2 5PT, UK,; ^2^Department of Molecular Pathology, The Institute of Cancer Research, Surrey SM2 5NG, UK,; ^3^Department of Medical Oncology, Vall d’Hebron University Hospital, Universitat Autònoma de Barcelona, Barcelona 08035, Spain,; ^4^Department of Immunology, Genetics and Pathology, Experimental and Clinical Oncology, University of Uppsala, Uppsala 78751 85, Sweden,; ^5^Department of Haematology and Medical Oncology, Biomedical Research Institute INCLIVA, University of Valencia, Valencia 46010, Spain and; ^6^Department of Cancer Therapeutics, The Institute of Cancer Research, Surrey SM2 5NG, UK

## Abstract

Single nucleotide polymorphisms (SNPs) in microRNA genes have been associated with colorectal cancer (CRC) risk, survival and response to treatment. Conflicting results are available on the association between rs4919510, a SNP in mature miR-608 and clinical outcome in CRC. Here, we analyzed the association between rs4919510 and benefit from perioperative treatment in a randomised phase II trial of neoadjuvant Capecitabine and Oxaliplatin (CAPOX) followed by chemo-radiotherapy, surgery and adjuvant CAPOX ± Cetuximab in high-risk locally advanced rectal cancer (LARC). A total of 155/164 (94.5%) patients were assessable. 95 (61.3%) were homozygous for CC, 55 (35.5%) heterozygous (CG) and 5 (3.2%) homozygous for GG. Median follow-up was 64.9 months. In the CAPOX arm the 5-year progression-free survival (PFS) and overall survival (OS) rates were 54.6% and 60.7% for CC and 82.0% and 82.1% for CG/GG, respectively (HR PFS 0.13, 95% CI: 0.12–0.83, *P* = 0.02; HR OS 0.38, 95% CI: 0.14–1.01, *P* = 0.05). In the CAPOX-C arm PFS and OS were 73.2 and 82.2%, respectively for CC carriers and 64.6 and 73.1% for CG/GG carriers (HR PFS 1.38, 95% CI: 0.61–3.13, *P* = 0.44; HR OS 1.34, 95% CI: 0.52–3.48, *P* = 0.55). An interaction was found between study treatment and rs4919510 genotype for both PFS (*P* = 0.02) and OS (*P* = 0.07). This is the first study investigating rs4919510 in LARC. The CC genotype appeared to be associated with worse prognosis compared to the CG/GG genotype in patients treated with chemotherapy and chemo-radiotherapy alone. Addition of Cetuximab to chemotherapy and chemo-radiotherapy in CC carriers appeared to improve clinical outcome.

## Summary

Analysis of a polymorphism in mature microRNA-608 (rs4919510) in rectal cancer patients enrolled in a randomized phase II clinical trial identified patient subpopulations who might benefit from the use of an intensified neo-adjuvant treatment strategy with Cetuximab.

## Introduction

Management of locally advanced rectal cancer (LARC) is largely based on a number of clinical–radiological factors identified on baseline staging. However, it is clear that patients with similar risk factors at baseline can have different outcomes and may require tailored treatment approaches and surveillance strategies (1). Unfortunately, no predictive/prognostic biomarkers are currently available in this setting to allow personalised approaches based on tumor biological aggressiveness or initial response to neoadjuvant treatment.

MicroRNAs are short, non-coding RNA sequences that regulate gene expression by targeting hundreds of mRNAs (2). A number of key cellular processes including proliferation, differentiation and response to anticancer treatments are influenced by this regulatory mechanism (3).

Single nucleotide polymorphisms (SNPs) in microRNA genes have been increasingly analysed for their functional implications. A single base pair change in the nucleoside sequence can affect microRNA biogenesis, processing, and target site binding. Indeed, microRNA-encoding genes are highly conserved and the high frequency of SNPs within microRNA genes supports the importance of their function (4). Growing evidence supports a link between SNPs in microRNA genes and colorectal cancer (CRC) risk, prognosis and drug response (5,6).

The *mir-608* gene is located on chromosome 10 (10q24.31): a G>C substitution (*SNP*, *rs4919510*) affecting the mature miR-608 sequence has been associated with clinical outcome and response to treatment in CRC patients. Although there is a general consensus about a potential interaction between *rs4919510* and treatment outcome, discordant findings on the association between miR-608 genotype and drug response have been observed, most likely due to the lack of proper patient selection, heterogeneous chemotherapy regimens and different ethnicity in these retrospective series (7–10).

Here we report the first study of the role of rs4919510 in a prospective randomised phase II trial (EXPERT-C) of neo-adjuvant Capecitabine and Oxaliplatin (CAPOX) followed by chemo-radiotherapy (CRT), surgery and adjuvant CAPOX compared with the same treatment plus Cetuximab in Magnetic Resonance Imaging (MRI)-defined, high-risk LARC (11).

## Materials and methods

### EXPERT-C trial design

LARC patients were classified as high risk and eligible for the EXPERT-C trial based on the presence of at least one of the following factors on high-resolution pelvic MRI: (a) predicted circumferential resection margin involvement (i.e. tumor within 1mm of mesorectal fascia), (b) T3 distal tumor (i.e. tumor at/below elevators and extending beyond the muscolaris propria), (c) T3c (i.e., extramural tumor invasion between 5 and 15mm) or T3d tumor (i.e. extramural tumor invasion ≥15mm), (d) T4 tumor (i.e. tumor penetrating to the surface of the peritoneum or adherent to/invading other structures/organs), (e) extramural vascular invasion (EMVI). After central randomisation (1:1 ratio) patients received neoadjuvant CAPOX (four cycles) followed by capecitabine-based CRT, surgery and adjuvant CAPOX (four cycles) (CAPOX arm) or the same treatment plus weekly Cetuximab (CAPOX-C arm).

The study was approved by local ethics committees and institutional review boards and written informed consent was obtained from each patient before study entry including consent for future research (ISRCTN registration: 99828560).

### SNP selection, genotyping and molecular analyses

Only patients who had tumor tissue available for genomic analysis were eligible for this study. We selected SNPs in microRNA genes and their binding sites previously associated with response to treatment in CRC (7,12) to test for a potential association with response to neoadjuvant chemotherapy and Cetuximab. Results on the association between let-7 complementary site 6 (*LCS6 KRAS* variant) and outcome in the EXPERT-C trial have previously been reported (13).

DNA was extracted from formalin-fixed paraffin-embedded tumor tissue (FFPE) from pre-treatment biopsy and/or post-treatment resection specimens using the QIAamp DNA FFPE Tissue Kit (Qiagen, Hilden, Germany) and from peripheral blood mononuclear cells (PBMC) with QIAamp DNA Blood Mini Kit on QIAcube (Qiagen) as we previously described (14). Samples were genotyped using the Taqman assay (Life Technologies, Carlsbad, CA) for rs4919510 in mir-608. Cases, negative controls, and duplicate samples were processed in a random order, with 10% duplicates to test both inter- and intra-plate concordance. All parties involved in genotyping were blinded to the clinical data. Both inter- and intra-plate duplicates were 100% concordant. Mutational analyses of *KRAS* (exons 2–4), *NRAS* (exons 2–4), *BRAF* (codon 600) and *TP53* (exons 4–9) were performed centrally on genomic DNA as we previously described (15,16).

### Statistical analysis

The Chi-square test was used to assess whether the *mir-608* genotypes in the study population were in Hardy–Weinberg equilibrium. Progression-free survival (PFS) was defined as the time between randomization and tumor progression or death. Patients alive and without evidence of tumor progression at the time of the analysis were censored at last follow-up. Overall survival (OS) was defined as the time between randomization and death (or censored at last follow-up for patients who were alive at the time of the analysis). The Kaplan-Meier method was used to calculate survival estimates, and comparison of the treatment arms was carried out using a log-rank analysis. Hazard ratios and 95% confidence intervals were obtained from Cox regression. An interaction term between treatment arm and *mir-608* genotype was included in the Cox regression to test for a significant interaction. Multivariate Cox regression was used to assess whether a significant interaction remained significant after addition of prognostic variables. Prognostic variables including sex, World Health Organization (WHO) performance status at baseline (0 versus ≥1), baseline T stage (T4 versus other), TNM stage (stage II versus stage III) baseline mrEMVI, *RAS* status (wild-type versus mutant) and *TP53* status (wild-type versus mutant) were included in the multivariate models using forward selection if *P* value in univariate analyses was <0.1.

## Results

Genotyping of the *rs4919510* locus was performed on DNA extracted from pre (*n* = 113) and post (*n* = 122) neo-adjuvant treatment FFPE biopsies in 155 out of 164 eligible patients enrolled in the EXPERT-C trial: this cohort represents 94.5% and is representative of the trial population as we have previously shown (15) (tumor blocks were not available in the remaining cases). The same analysis was carried out in 105 pre-treatment matching peripheral blood samples (64% of the trial population; blood was not available in the remaining cases).

Eighty-one cases had both pre and post treatment biopsies: in this group the concordance rate between pre and post-chemotherapy genotyping was 98.7%. One discordant case showed GC genotype in the pretreatment biopsy and GG genotype in the resection specimen. In this case it is possible that neoadjuvant treatment might have altered the genotype but given that all the survival outcomes were calculated from randomization, only the pretreatment genotype was used in our analysis. DNA isolated from tumor tissues has been widely used for pharmacogenomic studies; the concordance rate between frequencies of SNPs in tumors and their matching bloods appears quite high with discordance rate lower than 1.5% (17).

In order to rule out any bias due to somatic alterations (such as loss of heterozygosity) that might have affected genotyping in FFPE versus bloods, we compared bloods and tumor tissues in the 101 patients for whom both materials were available and we observed 100% concordance. Interestingly the case with discordant findings between pre and post-treatment tumor samples showed concordance of results between pre-treatment tumor sample and blood, thus supporting our decision to use the pre-treatment genotype for analysis. Based on this observation we performed all our survival analyses on data obtained from FFPE material in order to increase the statistical power of the study.

Sixty patients (38.7%) were found to carry the polymorphic variant and these were evenly distributed between the two treatment groups (Table 1). The frequency of the miR-608 genotypes did not deviate from the Hardy–Weinberg equilibrium (*P* = 0.379) and no significant association was observed between genotype and baseline clinical–pathological characteristics or mutations in the *KRAS, NRAS, BRAF, PIK3CA* and *TP53 gene.* (Table 2).

**Table 1.** miR-608 genotype in the entire study population and by treatment arm

miR-608 genotype	CAPOX	CAPOX-C	All patients
	*N* (%)	*N* (%)	*N* (%)
CC	50 (64.1)	45 (58.4)	95 (61.3)
CG	25 (32.1)	30 (39.0)	55 (35.5)
GG	3 (3.8)	2 (2.6)	5 (3.2)

CAPOX, Capecitabine + Oxaliplatin; CAPOX-C, Capecitabine + Oxaliplatin + Cetuximab.

**Table 2.** Baseline patient characteristics by mir-608 genotype and treatment arm

	CAPOX (*n* = 78)		CAPOX-C (*n* = 77)	
	CC (*n* = 50) (%)	CG/GG (*n* = 28) (%)	CC (*n* = 45) (%)	CG/GG (*n* = 32) (%)
Gender				
Male	27 (54.0)	17 (60.7)	29 (64.4)	21 (65.6)
Female	23 (46.0)	11 (39.3)	16 (35.6)	11 (34.4)
Age (years)				
Median (range)	66 (28–79)	64 (35–75)	59 (31–75)	60 (35–75)
WHO PS				
0	23 (46.0)	14 (50.0)	25 (55.6)	11 (34.4)
≥1	27 (54.0)	14 (50.0)	20 (44.4)	21 (65.6)
MRI high-risk features				
T3c–T3d (≥5mm)	36 (72.0)	18 (64.3)	23 (51.1)	21 (65.6)
T4	11 (22.0)	8 (28.6)	14 (31.1)	6 (18.8)
CRM+/at risk	28 (56.0)	17 (60.7)	26 (57.8)	18 (56.3)
EMVI positive	37 (74.0)	21 (75.0)	32 (71.1)	24 (75.0)
Low lying tumor	34 (68.0)	20 (71.4)	31 (68.9)	29 (90.6)
Tumor mutations				
*KRAS*	21 (42.0)	11 (39.3)	17 (37.8)	17 (53.1)
*NRAS*	2 (4.0)	2 (7.1)	2 (4.4)	0
*KRAS/NRAS*	23 (46.0)	13 (46.4)	19 (42.2)	17 (53.1)
*BRAF*	0	2 (7.1)	0	2 (6.3)
*PI3KCA*	6 (12.0)	1 (3.6)	3 (6.7)	0
*TP53*	24 (48.0)	11 (39.3)	20 (44.4)	20 (62.5)

CAPOX, Capecitabine + Oxaliplatin; CAPOX-C, Capecitabine + Oxaliplatin + Cetuximab; PS, Performance Status; WHO, World Health Organization.

After a median follow-up of 64.9 months (95% CI: 62.8–67.2), no statistically significant differences in PFS [63.5% (95% CI: 53.7–73.3) versus 72.9% (95% CI: 61.5–84.3) at 5 years; HR 0.67 (95% CI: 0.37–1.21) *P* = 0.18] and OS [71.0% (95% CI: 61.8–80.2) versus 77.6% (95% CI: 66.8–88.4) at 5 years, HR 0.66 (95% CI: 0.34–1.26), *P* = 0.208] were observed in the overall population between patients homozygous for the C allele and those carrying the G allele.

**Figure 1.**
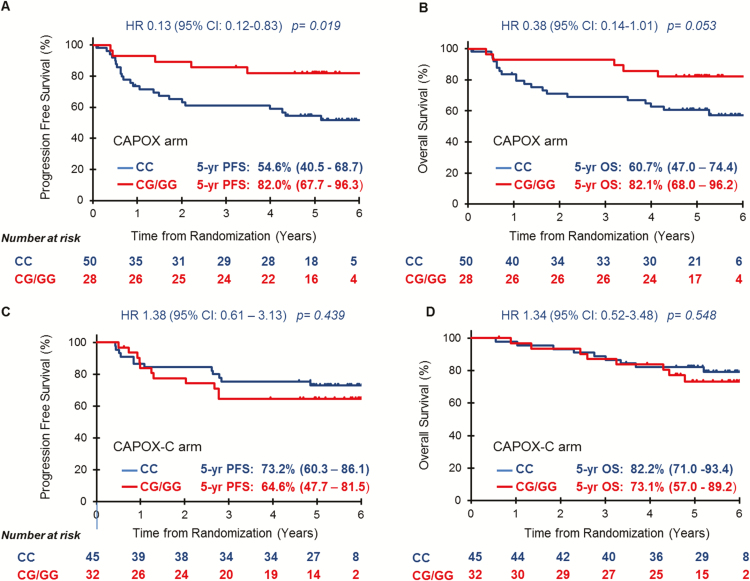
Survival outcomes according to miR-608 genotype in the study population. Progression-free survival (**A**) and overall survival (**B**) in the CAPOX (Capecitabine + Oxaliplatin) arm; progression-free survival (**C**) and overall survival (**D**) in the CAPOX-C (Capecitabine + Oxaliplatin + Cetuximab) arm. CI, confidence interval; HR, hazard ratio; PFS, progression-free survival; OS, overall survival.

In the CAPOX arm, patients with the CC genotype had worse 5-year PFS [54.6% (95% CI: 40.5–68.7) versus 82.0% (95% CI: 67.7–96.3) HR 0.13 (95% CI: 0.12–0.83) *P* = 0.019, adjusted *P* = 0.010] and 5-year OS [60.7% (95% CI: 47.0–74.4) versus 82.1% (95% CI: 68.0–96.2) HR 0.38 (95% CI: 0.14–1.01), *P* = 0.053, adjusted *P* = 0.033 (adjusted for p53 mutations (15)] compared to patients with the variant genotype (Figure 1A and B). These findings are in line with the data observed by Pardini (7) and Xing (8), and confirm an association between CC genotype and poor outcome in CRC.

Conversely, no survival differences by genotype were observed in the group of patients who received Cetuximab in combination with chemotherapy and CRT. The 5-year PFS was 73.2% (95% CI: 60.3–86.1) in patients homozygous for the C allele and 64.6% (95% CI: 47.7–81.5) in patients carrying the G allele [HR 1.38 (95% CI: 0.61–3.13) *P* = 0.439]. In the same genotype groups, the 5-year OS rates were 82.2% (95% CI: 71.0–93.4) and 73.1% (95% CI: 57.0–89.2) [HR 1.34 (95% CI: 0.52–3.48) *P* = 0.548], respectively (Figure 1C and D).

**Figure 2.**
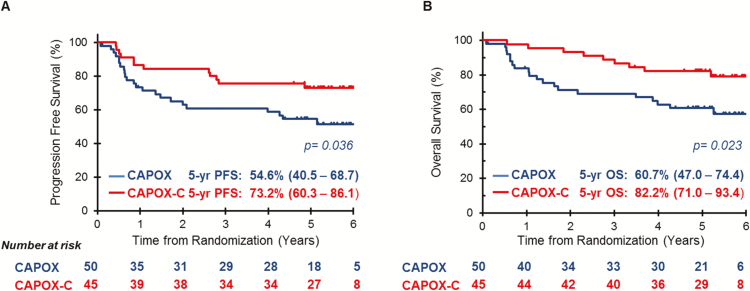
Survival outcomes in patients with the CC genotype according to treatment. Progression-free survival (**A**) and Overall survival (**B**). CAPOX, Capecitabine + Oxaliplatin; CAPOX-C, Capecitabine + Oxaliplatin + Cetuximab; CI, confidence interval; PFS, progression-free survival; OS, overall survival; HR, hazard ratio.

When we explored the effect of the addition of Cetuximab to chemotherapy and CRT in the CC carriers we noticed an improved 5-year PFS [73.2% (95% CI: 60.3–86.1) versus 54.6% (95% CI: 40.5–68.7) p: 0.036], and 5-year OS [82.2% (95% CI: 71.0–93.4) versus 60.7% (95% CI: 47.0–74.4), p: 0.023] (Figure 2A and B). The rate of complete responses in CC carriers was increased in the CAPOX-C arm (17.8%) versus the CAPOX arm (12.0) (Table 3), however the difference was not statistically significant possibly due to the small number of patients in the trial.

**Table 3.** Complete response by mir-608 genotype and treatment arm

miR-608 genotype	CAPOX (*n* = 78)	CAPOX-C (*n* = 77)	*P* value	All patients (*n* = 155)
	*N* (%)	*N* (%)		*N* (%)
**CC**	6/50 (12.0)	8/45 (17.8)	0.564	14/95 (14.7)
**CG/GG**	4/28 (14.3)	4/32 (12.5)	1.00	8/60 (13.3)
*P* value	0.740	0.751		0.807

CAPOX, Capecitabine + Oxaliplatin; CAPOX-C, Capecitabine + Oxaliplatin + Cetuximab.

In the CG/GG carriers, who appear to have a better prognosis compared to CC carriers, addition of Cetuximab to chemotherapy and CRT was not associated with any clinical benefit: 5-year PFS in the CAPOX arm was 82.0% (95% CI: 67.7–96.3) versus 64.6% (95% CI: 47.7–81.5) in the CAPOX-C arm [HR 2.17 (95% CI: 0.75–6.25), *P* = 0.152]; 5-year OS were 82.1% (95% CI: 68.0–96.2) and 73.1% (95% CI: 57.0–89.2), respectively [HR 1.48 (95% CI: 0.48–4.53), *P* = 0.492] (Supplementary Figure 1, available at *Carcinogenesis* Online). In contrast, combining Cetuximab with chemotherapy and CRT appeared to have a detrimental effect in carriers of the variant alleles, who were found to have an increased risk of distant relapse [Distant Relapse rate for the CAPOX-C arm 35.4% (95% CI: 18.5–52.3) versus 15.0% in CAPOX (95% CI: 1.5%–28.5%); HR 2.73 (95% CI: 0.87–8.59) *P* = 0.086] which translated into worse Distant Relapse Free Survival [CAPOX arm 85.0% (95% CI: 71.5–98.5) versus CAPOX-C 64.6% (95% CI: 47.7–81.5), HR 2.73 (95% CI: 0.87–8.59) with a trend towards a statistically significant interaction for distant tumor recurrence between treatment and *rs4919510* (*P* = 0.060; adjusted *P* = 0.092).

## Discussion

In this retrospective analysis of the EXPERT-C trial, we have shown that the *miR-608* CC genotype is associated with worse outcome when compared to the CG/GG genotypes in rectal cancer patients treated with neoadjuvant systemic chemotherapy followed by CRT, surgery and adjuvant chemotherapy. Interestingly, the addition of Cetuximab to this intensified treatment strategy seemed to rescue the poor prognosis of patients with the CC genotype. To our knowledge, our study is the first to evaluate the prognostic role of rs4919510 in a homogeneous cohort of rectal cancer patients. Our patient population was represented by a prospectively collected series of LARC patients with an extensive molecular characterisation.

Previous case-control studies have explored *miR-608* in relation to survival in CRC patients, with conflicting results. Pardini *et al.* (7) studied a European cohort of CRC patients and confirmed the presence of an association between rs4919510 and survival only in patients with stage III CRC who received 5-Fluorouracil (5-FU) based adjuvant chemotherapy. They observed that carriers of the G allele were at significantly decreased risk of recurrence when compared with CC genotype carriers. Similarly, Xing *et al.* (8) found that this SNP was associated with favourable outcome in 319 patients who received FOLFOX adjuvant chemotherapy, while this association was not evident in 89 patients who did not receive chemotherapy.

Contrary to these studies, Lin *et al.* (9) reported that the rs4919510 SNP was associated with poor outcome in stage III CRC receiving 5-FU based adjuvant chemotherapy. Several factors may account for the discrepancy between Lin’s findings and our observations: (i) both the training and the replication set in Lin’s study included 10% African-American (and 10% other ethnicities) in whom GG genotype was shown to be more frequent; (ii) while our cohort included only rectal cancer, Lin and colleagues studied a mixed population of proximal and distal colon and rectal cancers with the latter being less than 50%. Despite their analysis included adjustment for tumor site, the proportion of rectal cancer in the 179 stage III CRC patients is not specified.

As Ryan (4) and colleagues pointed out, ethnicity may account for important discrepancies in the prediction of CRC risk and prognosis among different populations: they found a significant association between the variant GG genotype and increased risk of death in Caucasian patients. Conversely, in African-American patients, there was a trend for the GG genotype to be associated with decreased risk of death, although this did not reach statistical significance (HR GG versus CC 0.38, 95% CI, 0.13–1.13, *P* = 0.082, adjusted HR GG versus CC 0.36, 95% CI 0.12–1.07, *P* = 0.66). Unfortunately the study did not include clinical information related to treatment, making comparison with our study challenging.

MicroRNA expression is tissue and organ specific and differences in microRNA deregulation have been observed in rectal compared to colon cancers (18–21). It is likely that the effects of *rs4919510* on the interaction between miR-608 and its wide spectrum of target mRNAs may also differ according to the location of the primary tumor, thus possibly explaining some divergent results observed among studies which included heterogeneous patient populations. Notably, as suggested by Ryan and colleagues, two genes involved in the fluoropyrimidine metabolism [thymidine kinase and folylpolyglutamate synthase (10,22)] have been included in the list of putative targets of miR-608 and may potentially account for the effects of *rs4919510* on the modulation of response to fluorouracil when given in combination with other chemotherapy drugs or as a radio-sensitizer with radiotherapy.

All patients included in our study received the same treatment with the exception of the addition of Cetuximab for those randomised to the investigational arm. This allowed us to explore for the first time the potential association between *rs4919510* and activity of this anti-epidermal growth factor (EGFR) monoclonal antibody. Notably, the administration of Cetuximab appeared to improve the outcome of patients with the CC genotype while no incremental benefit from its use was observed in the group of patients with the CG/GG genotypes. This resulted in a statistically significant interaction for survival between Cetuximab treatment and mir-608 genotype. These findings suggest that rs4919510 may potentially interfere with the mechanism of action of Cetuximab ultimately leading to an attenuation of its anti-tumor properties. In support of this theory, miR-608 has been reported to target the EGFR and other genes which were previously shown to mediate resistance to EGFR inhibition such as MET (23). Alteration of binding affinities of mir-608 to these targets may explain the absence of Cetuximab benefit in carriers of the G allele.

Even though our cohort has been prospectively collected and is homogeneous in term of ethnicity and treatment, we acknowledge that our study may have some limitations: (i) the analysis of *rs4919510* was not originally planned when the EXPERT-C study was designed and therefore it suffers from all the limitations inherent to retrospective biomarker analyses; (ii) Given the investigational nature of both treatment arms of the EXPERT-C trial, it is not known whether the study findings are applicable to a rectal cancer patient population treated with standard fluoropyrimidine-based CRT.

In conclusion, we believe that our findings are of interest and support the importance of small non-coding RNAs as potential determinants of tumor aggressiveness and/or response to treatment in LARC patients prompting further analysis in this setting. An extensive analysis of microRNA expression in patients from the same series is ongoing.

## Supplementary material

Supplementary Figure 1 can be found at http://carcin.oxfordjournals.org/

## Funding

The work was supported by Cancer Research UK (CEA A18052), European Union FP7 (CIG 334261) and the National Institute for Health Research (NIHR) Biomedical Research Centre (BRC) at The Royal Marsden NHS Foundation Trust and The Institute of Cancer Research (grant A62) to N.V.

## Supplementary Material

Supplementary Data

## Abbreviations

CRC: colorectal cancer
EMVI: extramural vascular invasion
LARC: locally advanced rectal cancer
OS: overall survival
PFS: progression-free survival
SNP: single nucleotide polymorphism
