# Supplementary material for: Sequence variation in mature microRNA-608 and benefit from neo-adjuvant treatment in locally advanced rectal cancer patients
Source: Carcinogenesis. 2016 Jul 5;37(9):852–7. doi: 10.1093/carcin/bgw073 (PMC5008250; doi:10.1093/carcin/bgw073)
Supplement: Supplementary Data [file supp_37_9_852__index.html]

Sequence Variation in Mature MicroRNA-608 and benefit from neo-adjuvant treatment in locally advanced rectal cancer patients — Sequence variation in mature microRNA-608 and benefit from neo-adjuvant treatment in locally advanced rectal cancer patients — Sequence variation in mature microRNA-608 and benefit from neo-adjuvant treatment in locally advanced rectal cancer patients — Supplementary Data 

# Sequence variation in mature microRNA-608 and benefit from neo-adjuvant treatment in locally advanced rectal cancer patients

## Supplementary Data

Data files

- Supplementary Data - Supplementary Data
