# Supplementary figures and images for: Sequence variation in mature microRNA-608 and benefit from neo-adjuvant treatment in locally advanced rectal cancer patients
Source: Carcinogenesis. 2016 Jul 5;37(9):852–7. doi: 10.1093/carcin/bgw073 (PMC5008250; doi:10.1093/carcin/bgw073)

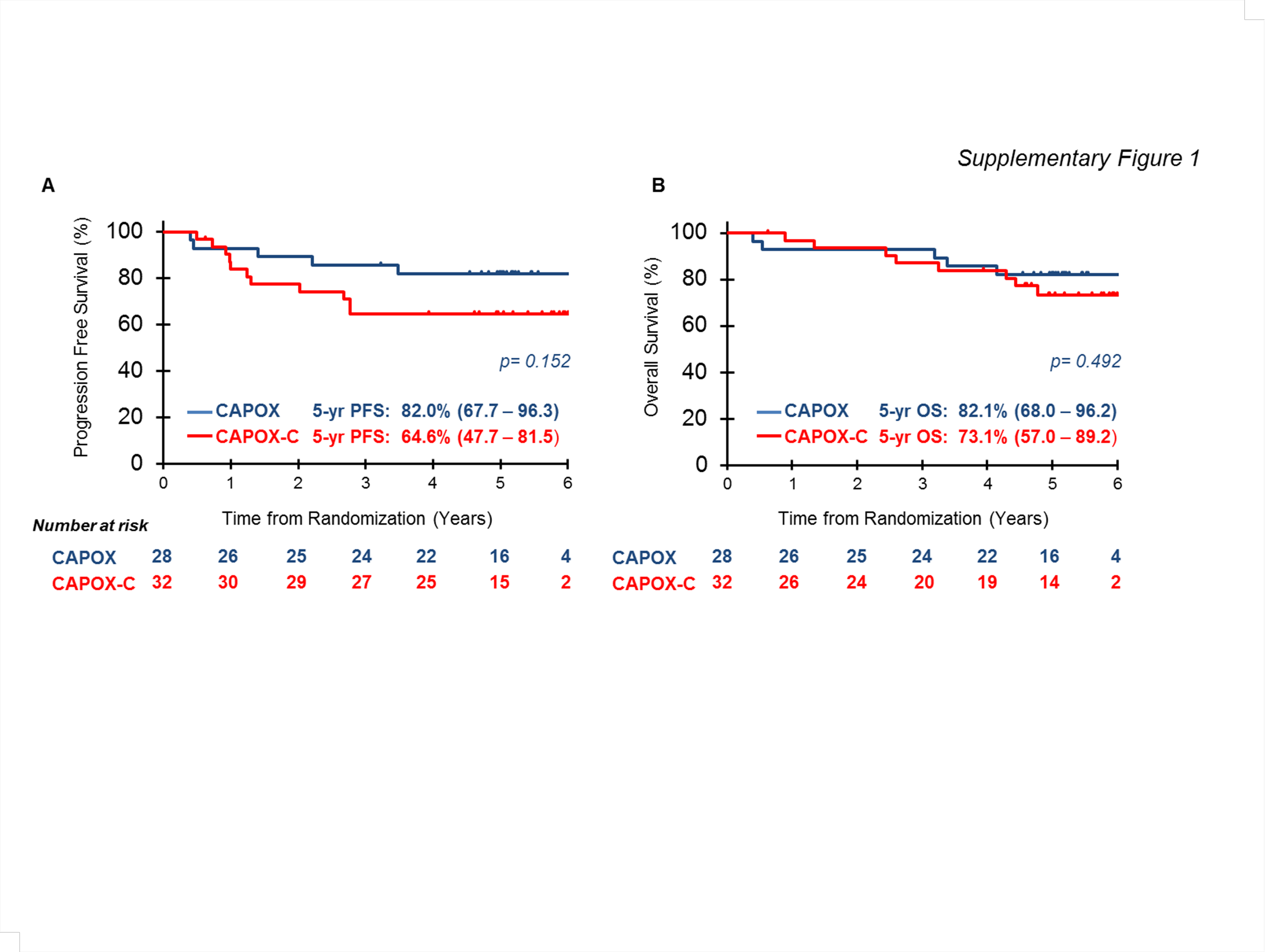

Supplement: Supplementary Data [file supp_bgw073_Picture3.tif]
